# Photochemical and Oxidative Degradation of Chamazulene Contained in *Artemisia*, *Matricaria* and *Achillea* Essential Oils and Setup of Protection Strategies

**DOI:** 10.3390/molecules29112604

**Published:** 2024-06-01

**Authors:** Simone Gabbanini, Jerome Ngwa Neba, Riccardo Matera, Luca Valgimigli

**Affiliations:** 1 R&D Department, BeC s.r.l., Via C. Monteverdi 49, 47122 Forlì, Italy; ricerca@bec-natura.com; 2 Department of Chemistry “Ciamician”, University of Bologna, Via Gobetti 85, 40129 Bologna, Italy; jeromengwa.neba@studio.unibo.it; 3 Tecnopolo di Rimini, Via D. Campana 71, 47922 Rimini, Italy

**Keywords:** *Artemisia arborescens*, blue color, analysis, UHPLC-MS/MS, GC-MS, photodegradation, antioxidant, sunscreen

## Abstract

Chamazulene (CA) is an intensely blue molecule with a wealth of biological properties. In cosmetics, chamazulene is exploited as a natural coloring and soothing agent. CA is unstable and tends to spontaneously degrade, accelerated by light. We studied the photodegradation of CA upon controlled exposure to UVB-UVA irradiation by multiple techniques, including GC-MS, UHPLC-PDA-ESI-MS/MS and by direct infusion in ESI-MS^n^, which were matched to in silico mass spectral simulations to identify degradation products. Seven byproducts formed upon UVA exposure for 3 h at 70 mW/cm^2^ (blue-to-green color change) were identified, including CA dimers and CA benzenoid, which were not found on extended 6 h irradiation (green-to-yellow fading). Photostability tests with reduced irradiance conducted in various solvents in the presence/absence of air indicated highest degradation in acetonitrile in the presence of oxygen, suggesting a photo-oxidative mechanism. Testing in the presence of antioxidants (tocopherol, ascorbyl palmitate, hydroxytyrosol, bakuchiol, γ-terpinene, TEMPO and their combinations) indicated the highest protection by tocopherol and TEMPO. Sunscreens ethylhexyl methoxycinnamate and particularly Tinosorb^®^ S (but not octocrylene) showed good CA photoprotection. Thermal stability tests indicated no degradation of CA in acetonitrile at 50 °C in the dark for 50 days; however, accelerated degradation occurred in the presence of ascorbyl palmitate.

## 1. Introduction

**Scheme 1:**
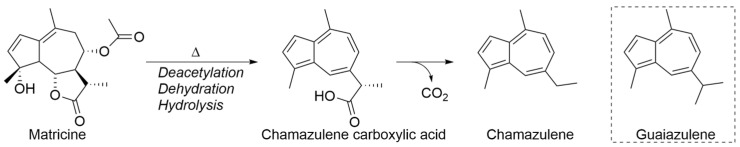
Decomposition of matricin to CA via chamazulene carboxylic acid, showing guaiazulene structure for comparison.

Chamazulene, CA (1,4-dimethyl-7-ethylazulene) is a bicyclic unsaturated hydrocarbon with the molecular formula C_14_H_16_. It is found in essential oils (EOs) from a variety of plants [1,2,3,4], notably German chamomile (*Matricaria recutita*, L.) [5], wormwood (*Artemisia absinthium*, L.) [6] and yarrow (*Achillea millefolium*, L.) [7]. CA is a blue-violet derivative of azulene formed by the thermal decomposition of colorless sesquiterpene matricin during the steam distillation process [8] (Scheme 1). Matricin is a genuine compound of, e.g., chamomile that is unstable under acid conditions. Upon steam distillation, it is readily converted into chamazulene carboxylic acid and further into chamazulene. Its deep blue color is a main feature determining its interest and the value of EOs containing it. Besides being used as a natural colorant in cosmetics and pharmaceuticals, chamazulene has anti-inflammatory activity [1] and has been proven to slow down the oxidation of cumene [9], and it has been used to protect human dermal fibroblast from oxidation by reactive oxygen species (ROS), specifically by boosting the biosynthesis of antioxidant enzymes, which would break down ROS present in the medium [10]. Hence, CA has indirect antioxidant properties, besides showing reactivity toward different radicals [11]. EOs are well-perceived natural sources of bioactives of widespread use in cosmetics and food products [12,13], and CA has gained popularity as an ingredient in cosmetic skin- and hair-care products, also owing to its reported photoprotective activity, making it able to prevent UVB-induced photodamage [14].

Paradoxically, this last property might actually turn into a matter of concern. While people using these products are unavoidably exposed to sunlight, it is not known whether the use of cosmetics containing CA with concomitant exposure to sunlight results in any adverse effects. This has made it vital to carry out an investigation of the photostability of CA. Besides its toxicological implications, the fading away of natural organic dyes such as azulene derivatives on exposure to ambient or normal storage conditions is a main issue limiting their usefulness and the stability of cosmetics and health-related products [15], as degradation intermediates could also trigger the oxidative damage of other sensitive components, such as lipids [16].

Although there are studies regarding the (limited) stability of related guaiazulene in cosmetic formulations and under conditions of exposure to light and high temperatures [15,17], knowledge on CA appears scarce, despite its similarity to guaiazulene and its importance. Indeed, guaiazulene, differing from CA by an isopropyl substituent in the ring (Scheme 1), is perhaps a better-known yet a less frequent component of EOs, sharing similar pharmacological properties [18,19]. While the two azulenes can sometimes be found in the same EO, e.g., of *M. chamomilla* L. [20], the most frequent and typical (often the only) dye found in “blue chamomile” EO is actually CA [21,22,23]. In the present study, CA was isolated from *Artemisia arborescens* (L.) EO, obtained by hydrodistillation from wild specimens from Sicily (Italy). Its thermal and photochemical stability in the presence and absence of oxygen were investigated, since studies have shown that oxygen is often involved in the instability of organic dyes [24,25]. This work aims at achieving a mechanistic understanding of the photo- and oxidative degradation pathways of CA using kinetic approaches and different analytical techniques to identify byproducts. Since our aim was also as to propose prevention strategies, we investigated the use of antioxidants and sunscreens [26] to protect CA under photochemical and thermal stress. 

## 2. Results and Discussion

### 2.1. CA Isolation and Purification

**Figure 1:**
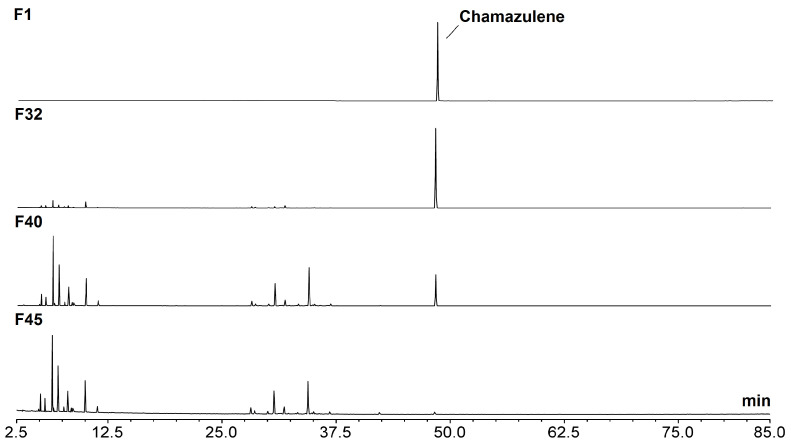
Total ion current GC-MS chromatogram of different fractions of *Artemisia arborescens* (L.) essential oil obtained by flash chromatography; see Figure S1 for whole EO profile. Fractions F1 to F31 contained > 99% chamazulene, while the other fractions with variable impurities were discarded.

CA was isolated by normal phase flash chromatography from *Artemisia arborescens* (L.) essential oil. After comparative screening with different *M. chamomilla* and *A. millefolium* EO specimens (see Figure S1 in Supplementary Materials), this essential oil was chosen as the most convenient source for three main reasons: (1) it is very rich in CA (often >10%), (2) it is relatively inexpensive because the plant grows spontaneously in many Mediterranean areas and has limited commercial interest, and, finally, (3) it allows for easy isolation because in *A. arborescens* essential oil, CA is the most lipophilic component. The sample we used contained 13.03% CA by GC-MS analysis. Thus, 2.5 g of EO was seeded on a flash chromatography silica column and eluted with hexane; the blue band of CA was collected in fractions whose purity was monitored by TLC. A better purity evaluation was performed after collection by GC-MS. The fractions with a GC-MS peak area larger than 99% were combined and dried to afford CA with a purity of 99.8%, which was used for subsequent studies. Approximately 138 mg of pure CA were obtained with an isolation yield of 5.52% (Figure 1).

### 2.2. Generation of Photo-Degradation Products of CA

Our first aim was to identify the products formed from CA photodegradation, so as to achieve understanding of the process. Acetonitrile solutions of CA at concentrations of 1000 mg/L were placed into quartz cells closed with a screwcap. The quartz cells were exposed to a W-Hg solar lamp emitting light in the Vis-UVA-UVB region (see Figure S2 in Supplementary Materials), at a distance from the light source to achieve an irradiance of 70 mW/cm^2^ in the UVA (strong photooxidation conditions) and were irradiated until visible color changes were obtained. 

**Figure 2:**
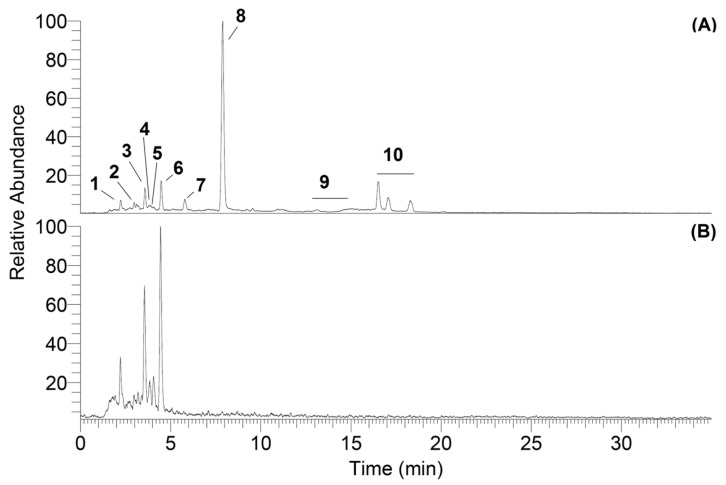
LC-MS chromatograms of photoexposed CA in acetonitrile: (**A**) green sample (exposure time: 3 h); (**B**) yellow sample (exposure time: 6 h). Chromatographic conditions: LC-MS method A (see Section 3.4).

A main change from a deep blue color to green was achieved after an exposure time of 3 h, then a complete fading to a yellow solution was obtained with an exposure time of 6 h. Therefore, the two solutions photoexposed for 3 h and 6 h were subjected to UHPLC-ESI-MS^n^ analysis. Typical chromatograms are shown in Figure 2. The green sample, still containing a CA residue, was richer in chromatographic peaks than the yellow sample, in which only three had survived and become dominant (Figure 2). 

Based on the studies by Matsubara et al. on the degradation of guaiazulene [27], to each peak characterized by a specific *m/z* ratio was assigned a hypothetical chemical structure that was then confirmed through MS^n^ experiments on the isolated parent ions, which were also performed by direct infusion of the sample into the ESI source. To aid in identification, the MS^n^ fragmentation tree obtained experimentally was matched with one generated in silico using Mass Frontier 5.0, a software program based on fragmentation libraries, which can predict the fragmentation pathway of a compound, following different ionization methods [28]. 

**Scheme 2:**
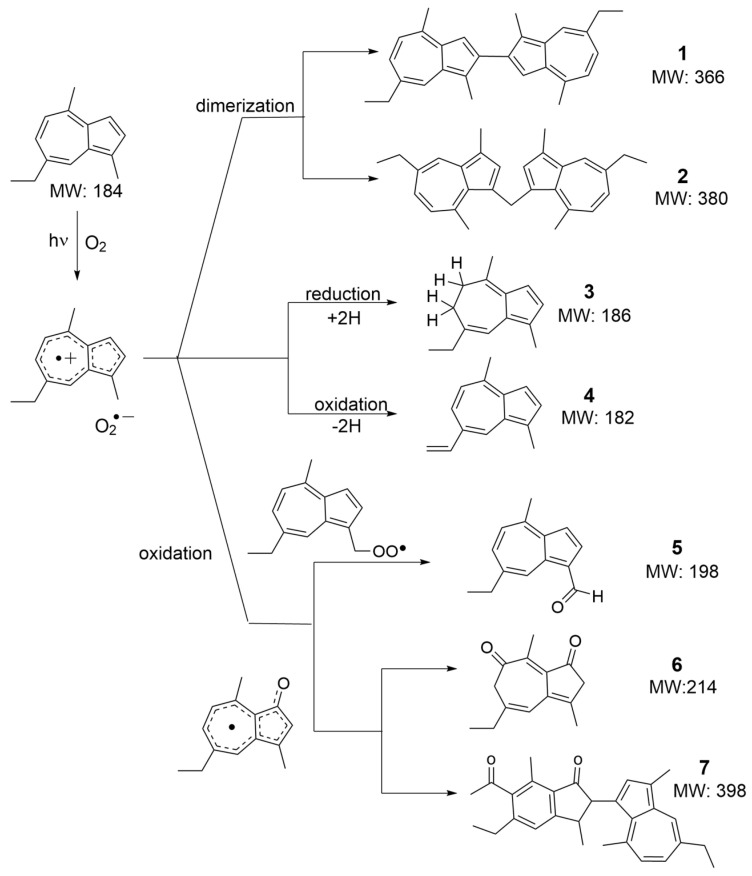
Reaction pathways for the formation of photo-oxidative products of CA. **1**: CA dimer, **2**: Methylene dimer of CA, **3**: Dihydrochamazulene, **4**: Oxidized CA, **5**: CA carbaldehyde, **6**: CA quinone and **7**: CA benzenoid.

This also provided the possible tandem mass spectrometry (MS/MS) fragment ions of an unknown compound [28]. The *m/z* of the fragment ions generated by the software were compared with mass spectra of the compound under investigation. This software was utilized to reduce the number of false positives. In addition, a bibliographic review of the possible chemical structures of the degradation products of chamazulene was performed, also by referencing to structurally related guaiazulene [17]. This combined approach enabled the identification of a significant number of photodegradation products present in the sample, despite the major complexity. The parent ions (*m/z*) and their product ions obtained by MS^n^ are reported in Table 1. Among those products we could identify with good confidence seven structures, which are illustrated in Scheme 2.

**Figure 3:**
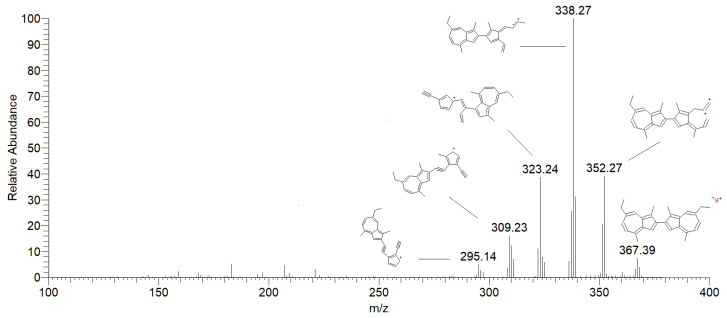
Mass spectra (MS/MS) showing the fragment ions of chamazulene dimer (**1**) parent ion [M + H]^+^ *m/z* 367 in tandem mass mode, by collision-induced dissociation (CID).

**Figure 4:**
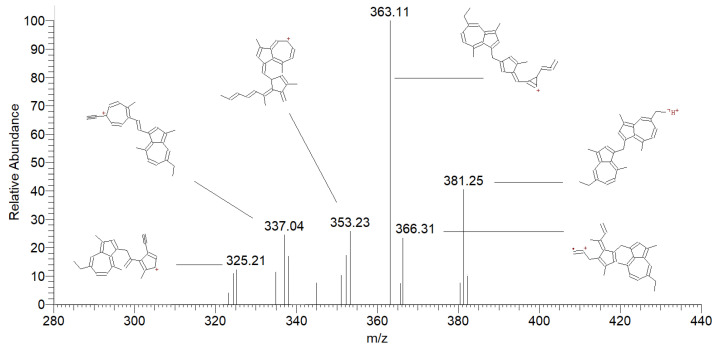
Mass spectra (MS/MS) showing the fragment ions of the methylene dimer of chamazulene (**2**) parent ion [M + H]^+^ *m/z* 381 acquired in tandem mass mode by CID.

With reference to Scheme 2, LC-MS analysis of the sample exposed to UV radiation for 3 h revealed the presence of dimers (**1** and **2**). Instead, in the sample solution irradiated for 6 h dimers were not found, based on the mass spectra. These products are formed when hydrogen is abstracted from chamazulene, giving rise to a radical that further combines with itself to form various dimers of chamazulene. Similar outcomes were obtained from studies investigating the photochemical degradation products of guaiazulene and azulene under UV light [17,29]. Besides dimers, more complex products, including oxygenated trimers, tetramers and even oligomers, were formed [17]. Also, it was found that the formation of 3,3-biguaiazulene (a dimer) in solution resulted in a color change from blue to green [29]. Accordingly, after 6 h of irradiation of our samples, when the color turned from green to yellow, dimers were no longer present, which indicates that the dimers formed in our sample were responsible for the green color. Therefore, further exposure to UV irradiation leads the decomposition of the dimers to form other products. Figure 3 and Figure 4 represent the mass spectra of CA dimer and the methylene dimer of CA, respectively, acquired in tandem mass mode. 

**Figure 5:**
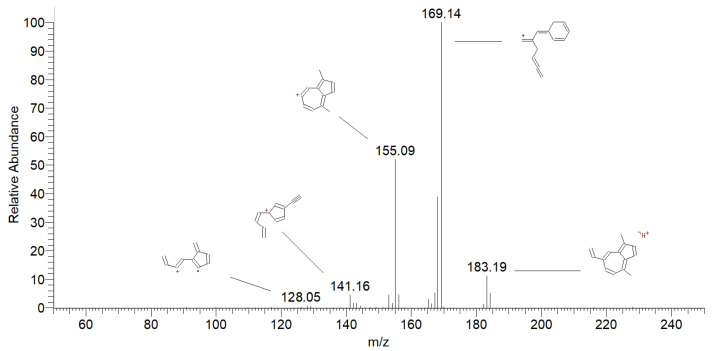
Mass spectra (MS/MS) showing the fragment ions of oxidized chamazulene (**3**) parent ion [M + H]^+^ with *m/z* 183 acquired in tandem mass mode by CID.

**Figure 6:**
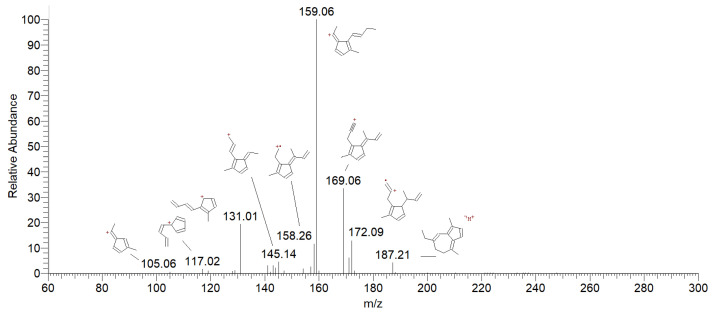
Mass spectra (MS/MS) showing the fragment ions of dihydrochamazulene (**4**) parent ion [M + H]^+^ with *m/z* 187 acquired in tandem mass mode by CID.

Degradation products such as dihydrochamazulene (*m/z* 186) and oxidized CA (*m/z* 182), as seen in Scheme 1, result from a disproportionation reaction of CA (*m/z* 184). They differ from each other by 2 mass units, which asserts the reduction of chamazulene (by the addition of 2 hydrogen atoms) for dihydrochamazulene and, on the other hand, the oxidation of chamazulene (by the loss of 2 hydrogen atoms). The ability of CA to undergo hydrogen abstraction by radical species has been reported in the literature as the suggested mechanism for its antioxidant activity [11]. One possible explanation is that excited CA serves as H-abstracting species towards ground-state CA, triggering the disproportionation. On the other hand, it was demonstrated by EPR spectroscopy that UVA irradiation of azulene and guaiazulene in the presence of oxygen forms superoxide radical (O_2_^−•^) by electron transfer from the photoexcited azulene [30]. Likely, a subsequent proton transfer (ET-PT sequence) or a concerted PCET (proton-coupled-electron-transfer) to O_2_ [31,32] would afford the hydroperoxyl radical HOO• (the neutral form of superoxide) and the C-centered radical of CA, which can decay by disproportionation. This last mechanism calls for the key role of oxygen in the photodegradation of CA. Figure 5 and Figure 6 represent the mass spectra of oxidized CA and dihydrochamazulene, respectively, acquired in tandem mass mode.

**Figure 7:**
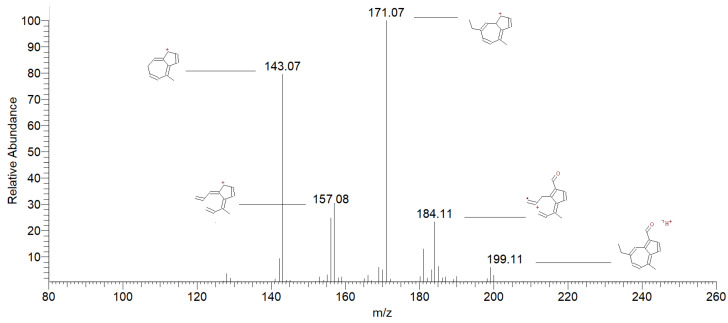
MS/MS spectrum showing the fragment ions of chamazulene carbaldehyde (**5**) parent ion [M + H]^+^ with *m/z* 199 acquired in tandem mass mode by CID.

Another photodegradation product, CA carbaldehyde, is most probably formed from the reaction of molecular oxygen with a chamazulene (carbon-centered) radical to give rise to a peroxyl radical intermediate (ROO•, Scheme 1), which subsequently affords a further sidechain oxidation, forming the aldehyde derivative of chamazulene. This manner of reaction is similar to the well-accepted mechanism of autooxidation of the sidechain in alkylbenzenes and alkylnaphthalenes [29,33,34]. The peroxyl radical is quite stable, yet highly reactive toward unsaturated lipids: when formed in a cosmetic product or other man-directed preparations, it can trigger the oxidation of other ingredients, causing the formation of toxic byproducts [16] and the formulation to lose its stability and change its properties. Additionally, Matsubara et al. [29] and Fiori et al. [15,17] both studied the photooxidation of alkyl azulenes in the presence and absence of oxygen and concluded that oxygen plays a vital role in the degradation of azulenic compounds. Figure 7 represents the mass spectrum of chamazulene carbaldehyde, acquired in tandem mass mode.

CA quinone and CA benzenoid (Scheme 2) are possibly formed through an alkoxyl radical intermediate, likely arising from hydroperoxyl (HOO•) or alkylperoxyl (ROO•, e.g., guaiazuleneperoxyl) radical addition to guaiazulene followed by fragmentation. Rearrangement of the conjugated structure can lead to quinone end products. In the case of the CA benzenoid, there is a complete modification of one azulene ring into a benzenoid.

**Figure 8:**
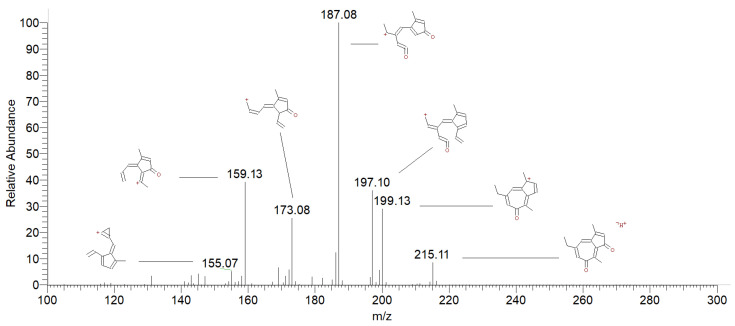
MS/MS spectrum showing the fragment ions of chamazulene quinone (**6**) parent ion [M + H]^+^ *m/z* 215 acquired in tandem mass mode by collision-induced dissociation.

It has been reported that the formation of degradation products that possess a modified azulene chromophore induced a color change in the sample solution [29]. This is because, as the azulene chromophore is modified, its spectrum of absorption in the visible would clearly change. The photochemical degradation pathway of CA resulting in quinone formation may hold significant implications for biological systems, depending on the site of exposure. At the skin level, similar byproducts of azulene and guaiazulene have been shown to cause various deleterious effects, including carcinogenicity, skin inflammation, and dermatitis [35,36]. Quinones can interfere with DNA synthesis, disrupting the normal transcription of information and consequently leading to the generation of mutated cells [37]. Figure 8 represents the mass spectra of CA quinone, acquired in tandem mass mode.

The fragmentation pathway of parent ion at *m/z* 215 obtained with Mass Frontier software is shown in Figure S3 (Supplementary Materials).

**Figure 9:**
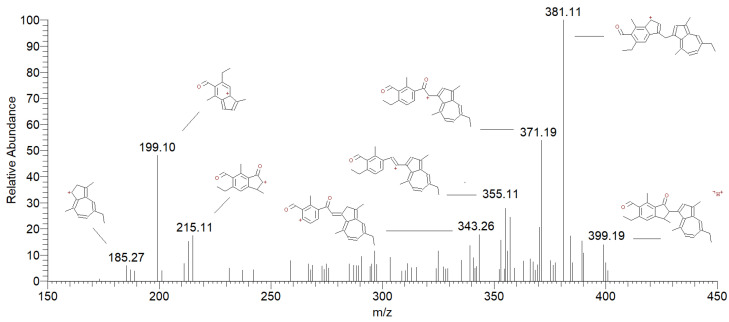
MS/MS spectrum showing the fragmentation of chamazulene benzenoid (**7**) parent ion [M + H]^+^ *m/z* 399 in tandem mass mode, by collision-induced dissociation.

Figure 9 represents the mass spectra of chamazulene benzenoid, acquired in tandem mass mode.

Overall, the photodegradation pathways identified here for CA are not dissimilar from those previously found for guaiazulene [17,29] although the actual structures and products’ distribution appear to hold some differences. These are conceivably to be attributed to the presence of the isopropyl group in guaiazulene. This enables the formation of a relatively stable benzyl-type tertiary alkyl radical in guaiazulene, which, in the presence of oxygen, would form a relatively persistent tertiary alkylperoxyl radical, having a different fate and reactivity compared to the primary or secondary alkyl/alkylperoxyl radicals attainable in CA [33,34].

### 2.3. Photostability Test

In order to evaluate the photostability of CA in solution under conditions that would be representative of the behavior in cosmetic products, we set out to operate according to ICH guidelines, option 1 [38], under weak photoirradiation conditions. Diluted (0.17 mM) solutions of CA were placed in closed quartz tubes at a distance from the light source to achieve an irradiance of 2.4 mW/cm^2^ in the UVA region. All experiments were performed by keeping a constant temperature (25 °C) and irradiance, as summarized in Table 2. Chamazulene concentration was monitored at regular time intervals (15, 30, 60, 120, 180, 240 min. corresponding at UVA irradiation doses of 2.2, 4.3, 8.6, 17.3, 25.9 and 34.6 Jcm^−2^, respectively) over 4 h of irradiation. Analysis was performed by GC-MS and by LC- PDA, upon setting suitable rapid methods allowing to monitor CA decay (Section 3.6 and Figures S4–S7 in Supplementary Materials). Concurrently, identical analyses were performed on samples stored in the dark (vials wrapped in aluminum foil), which served as controls. Otherwise identical sets of experiments were repeated by changing critical parameters so as to evaluate their influence on CA decay kinetics. Three solvents were comparatively tested to evaluate their influence on the kinetics of reaction and hopefully gather mechanistic insights: apolar hexane, protic methanol and polar acetonitrile, which was our reference solvent throughout the study. To evaluate the role of oxygen, samples were degassed by purging with nitrogen or left saturated with air with a variable volume of head space in the sealed tube.

Under identical experimental conditions, we also comparatively tested acetonitrile solutions of CA containing antioxidants (tocopherol, ascorbyl palmitate, TEMPO, hydroxytyrosol, bakuchiol, γ-terpinene) at concentrations up to 10 times that of chamazulene (1.7 mM) and sunscreens at 5% by weight, which corresponds to 0.2 mM, for octocrylene and octyl methoxycinnamate while it is 0.08 mM for and Tinosorb^®®^ S. These tests were meant to evaluate the protection that might be offered by other components of the cosmetic formulation [39] or the effectiveness of additives in slowing down photodegradation.

#### 2.3.1. Effect of Solvent and Oxygen

Solutions of 0.17 mM CA in acetonitrile, methanol and *n*-hexane, placed in a quartz EPR tube and photo-irradiated as reported in Table 2, showed different rates of degradation, highlighting a mechanism linked to the polarity of the solvent (Table 3). Indeed, in protic methanol, the rate of decay was twice as fast as in *n*-hexane, while it grew about 6 times faster in polar acetonitrile. Furthermore, it was sensitive to the head space (HS) volume of air in the tube, as can be judged from experiments in *n*-hexane carried out with the same irradiation but leaving a variable HS. The importance of oxygen is also highlighted by the much-reduced degradation recorded in N_2_-purged samples in any solvent. Indeed, while degradation became negligible in *n*-hexane, it reduced the rate about 3-fold in methanol and acetonitrile (Table 3).

Our results are in qualitative good agreement with previous studies on the photodegradation of azulene and guaiazulene by Matsubara et al. [29] and Fiori et al. [15,17] and strongly point toward a photo-oxidative path as the largely prevailing mechanism, with the formation of polar transition states and intermediates, stabilized by polar solvents. As a further confirmation of the photooxidative mechanism, a 500 mg/L solution of CA in acetonitrile was placed in a 3.5 mL Teflon sealed quartz cuvette and degassed for 5 min with an abundant N_2_ stream. In this way, it was possible to guarantee better degassing efficiency and complete air tightness. The cuvette was irradiated at 70 mW/cm^2^ (strong photooxidation conditions) for 6 h. At regular time intervals, a spectrophotometric reading was performed without opening the cuvette. Results shown in Figure S8 (Supplementary Materials) indicate a further 2-fold reduction of the rate of CA degradation compared to results in Table 3, despite the much hasher irradiation conditions.

#### 2.3.2. Effect of Antioxidants and Sunscreen

Since the photo-oxidative mechanism appeared dominant on the basis of previous experiments, we set out to evaluate the protection offered by antioxidants and sunscreens. The experiments were performed in acetonitrile, since it was the solvent affording the most marked and rapid photodegradation of CA, and solutions were saturated with air so as to simulate normal environmental conditions. Irradiance was set as in Table 2, both in the absence and in the presence of antioxidants and UV filters, and photodegradation was comparatively assayed by LC-PDA monitoring of CA concentration at 349 nm upon calibration (see Supplementary Materials). As shown in Table 4, in the absence of other additives, CA was completely degraded at the UVA irradiation dose of 35 J/cm^2^.

The selection of antioxidants deserves discussion. Our aim was to evaluate structurally different molecules of potential use in cosmetic formulas and possibly boosting different mechanisms. Besides α-tocopherol and ascorbyl palmitate, which are well-established highly effective chain-breaking antioxidants of major use in cosmetics [16,39], we tested bakuchiol, a potent natural phenolic antioxidant which is receiving major attention as a cosmetic ingredient, [40] and hydroxytyrosol, another natural cosmetic-grade and food-grade antioxidant which belongs to the catechol structural family [41]. In addition, we tested γ-terpinene, a terpene component of several essential oils that demonstrate an unconventional mechanism of inhibition, different from typical chain-breaking antioxidants, based on increasing the rate of decay of transient radical species [42,43]. The TEMPO (2,2,6,6-tetramethylpiperidine-*N*-oxyl) radical was also included, although it cannot be used as a cosmetic ingredient; however, it was used as a research molecule, as it boosts a unique antioxidant mechanism based on acting as a catalytic antioxidant in the presence of hydroperoxyl radicals [44]—these are possibly formed during the photodegradation of CA, as previously discussed.

Since antioxidants normally offer the best protection within synergic co-antioxidant mixtures [45,46], we also tested mixtures of the above antioxidants, including combinations that had previously been demonstrated to afford synergic activity [16,43,44]. All experiments are summarized in Table 4.

Disappointingly, most antioxidants and their combinations offered negligible protection to CA, underlying a clear difference between protecting a lipid from peroxidation radical chain and protecting an excited chromophore from photo-oxidative degradation under continuous irradiation. Indeed, some additives actually appeared to accelerate CA loss, likely due to photodegradation of the antioxidant itself, which would form radicals that might attack CA.

**Figure 10:**
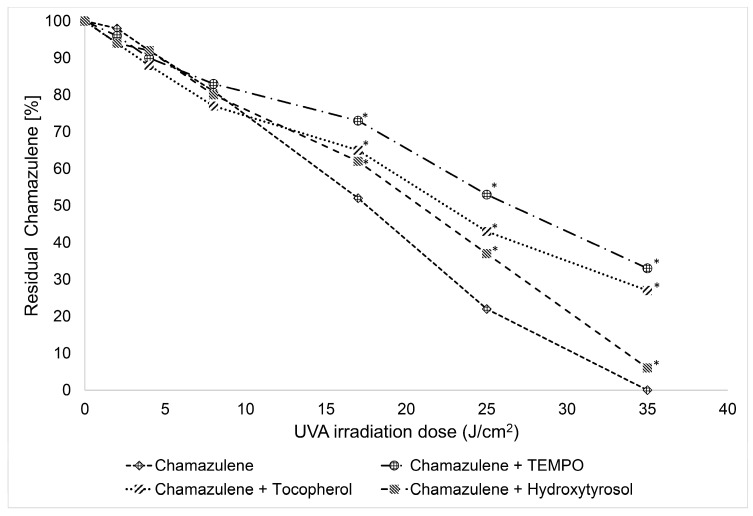
Photostability of CA (0.17 mM) in the presence of antioxidants (1.7 mM). Values significantly different (*p* > 0.01) from unprotected CA are marked by *.

However, some combinations afforded successful protection, slowing down the degradation of chamazulene to a significant extent. These are visually compared in Figure 10. Both hydroxytyrosol and even more so α-tocopherol effectively protected CA; however, the best protection was offered by TEMPO. Since it has been demonstrated that the antioxidant behavior of TEMPO is activated by hydroperoxyl radicals (HOO•) formed during the oxidation process [44], this intriguing observation apparently supports the mechanism we proposed to explain the formation of key degradation products like **3** and **4** (see Scheme 2 and Section 2.2), and, in general, it indicates that the intermediacy of the HOO•/O_2_^−•^ radical has a key role in the overall photo-oxidative degradation of CA. 

Experiments in the presence of sunscreens also offered interesting observations (Table 4). Three molecules were selected on the basis of their frequent occurrence in cosmetic products [47,48] and were comparatively tested: Tinosorb^®®^ S, a broad spectrum UVB-UVA filter (290–370 nm) with maxima of absorption at 310 and 350 nm, octocrylene, a UVB filter reaching the short-wavelength UVA region (280–330 nm, λ_max_ 307 nm) and octyl methoxycinnamate (OMC) with similar coverage (280–330 nm, λ_max_ 311 nm) [49,50,51].

**Figure 11:**
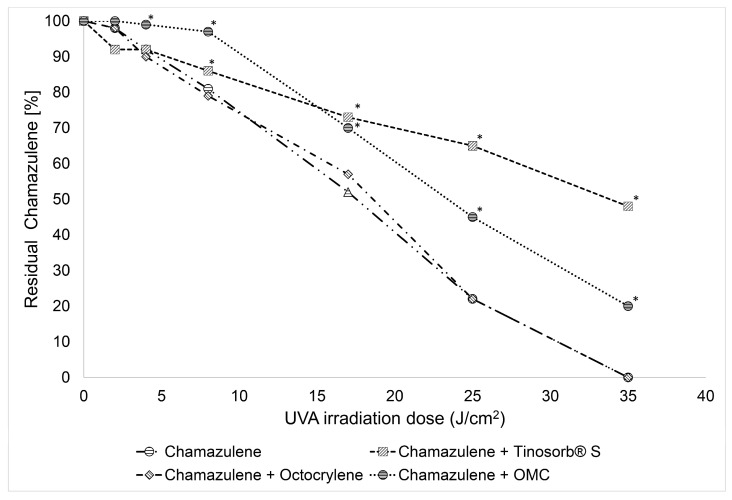
Photostability of chamazulene (0.17 mM) in the presence of UV filters at 5% concentration. Values significantly different (*p* > 0.01) from unprotected CA are marked by *.

Both Tinosorb^®^ S and OMC were able to effectively protect CA from photodegradation, as can be seen in Figure 11. Tinosorb^®^ S was overall the most effective, sparing as much as 48% of CA at the end of the experiment after prolonged exposure (4 h) to a cumulative irradiation dose of 35 J/cm^2^ in the UVA and 8 J/cm^2^ in the UVB. Nonetheless, OMC offered perhaps the most interesting result, as it completely blocked the loss of chamazulene for a lag time of about one hour, corresponding to an irradiation dose of about 10 J/cm^2^. Such a dose corresponds to 1-2 hours of exposure at noon on sunny days during the summer around the world, based upon observations of UVA intensity of 2.1 mW/cm^2^ in Okayama, Japan in September [52] and 3.6 mW/cm^2^ in Jackson (MS), USA in August [53]. However, following such a lag time, photodegradation started and proceeded at the same rate as the unprotected sample. This can tentatively be explained by considering that OMC itself has limited photostability [54], and it undergoes photodegradation when exposed to a solar lamp [55]; therefore, the end of the observed lag time could be attributed to its substantial photodegradation. Somewhat surprising was instead the lack of protection demonstrated by octocrylene, despite an absorption spectral range similar to OMC. Indeed, concerning their mechanism of photoprotection, all the tested sunscreens are expected to act by non-specific mechanism, i.e., by absorbing a relevant fraction of UVA-UVB radiation, whose energy is then dissipated in the form of heat, thereby reducing the effective incident UVA-UVB dose on CA, similarly to the known action on human skin [47,48].

Since also octocrylene is known to undergo degradation on exposure to UVA-UVB irradiation [56], possibly a similar explanation, as for the end of protection by OMC, can be put forward. Clearly, further studies monitoring the time course of OMC and octocrylene during irradiation would be needed to confirm this hypothesis. In the absence of direct irradiation, incubation of CA in the presence of each of the tested sunscreens in the dark or under ambient light for 24 h showed no reaction or degradation of CA or of any of the sunscreens, which apparently rules out specific interactions.

The efficacy exhibited by some antioxidants and UV filters in augmenting the photostability of chamazulene, at a concentration of typical use in cosmetics—both sunscreens are allowed at up to 10% (*w*/*w*), while antioxidants hydroxytyrosol and tocopherol are not limited [57]—signifies their potential application in formulations containing chamazulene to prevent its photooxidation when exposed to UV light, thus contributing to the overall stability and safety of the cosmetic formulation.

### 2.4. Thermal Stability of Chamazulene

**Figure 12:**
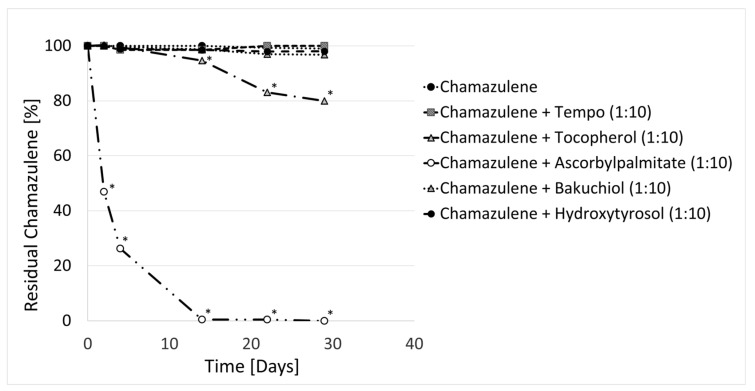
Thermal degradation of chamazulene in solution, alone or in the presence of antioxidants, incubated at 50 °C. Values significantly different (*p* > 0.01) from unprotected CA are marked by *.

In order to test the thermal stability of CA under conditions compatible with the storage and use of cosmetics, in the absence of direct light exposure, sample solutions of CA in acetonitrile were stored in the refrigerator at 4 °C, at room temperature and in the oven at 50 °C, all in the dark, for 50 days. Upon monitoring the concentration of CA over this period, it was observed that it remained stable throughout. Therefore, the stability of CA was not affected by the above-mentioned conditions. This is quite intriguing, since previous evidence suggests that cosmetic formulations containing essential oils rich in chamazulene tend to undergo color changes during prolonged storage in the dark at room temperature, implying that other processes not related to photodegradation would limit its stability. One possible explanation is that other labile components of the formulation might undergo oxidative degradation not triggered by light, and the radical or other intermediates formed during their oxidation or thermal degradation might attack CA, causing its degradation. As a proof of concept, we repeated the studies by monitoring the stability of CA in solution at different temperatures in the presence of antioxidants, which, by their nature, are very sensitive to oxidative thermal degradation. At room temperature or 4 °C, no sample showed any visible change or any measurable loss of CA, which indicates no direct reaction between CA and the tested antioxidants. However, results at 50 °C, summarized in Figure 12, were quite surprising. They show that, while CA alone was perfectly stable even after prolonged storage at 50 °C, the presence of tocopherol and, particularly, of ascorbyl palmitate accelerated its decay significantly, while other antioxidants did not produce a significant action in this regard, as shown in detail in Figure S9 (Supplementary Materials). This apparently paradoxical behavior is likely due to the high persistence of radicals formed by these two antioxidants [16], which reside in the sample sufficiently long to cause antioxidant-mediated autoxidation of CA. This parallels the well-known phenomenon of TMP, tocopherol-mediated peroxidation of human LDL caused by tocopherol, under some experimental settings [58]. As also discussed for TMP, the phenomenon can be completely abolished by using synergic mixtures of antioxidants in place of a single molecule. An issue that would require further investigation in cosmetic formulations, to assess its relevance and ways for prevention.

## 3. Materials and Methods

### 3.1. Materials

HPLC grade water, methanol and acetonitrile, formic acid and hexane (GC grade) were from Merck (Milan, Italy). Ascorbyl palmitate (ACEF, Piacenza, Italy), α-tocopherol (Artchem, Milan, Italy)), hydroxytyrosol (99%, Pioneer-biotech, Xian, China), bakuchiol (99%, Carlo Sessa S.p.A., Milan, Italy), (2,2,6,6-Tetramethylpiperidin-1-yl)oxyl (TEMPO) (98%, Sigma-Aldrich, Milan, Italy), octyl methoxycinnamate (98%, Sigma-Aldrich, also called ethylhexyl methoxycinnamate), bis-ethylhexyloxyphenol methoxyphenyl triazine (Tinosorb^®^ S, 99%, Eurotrading, Padova, Italy), octocrylene (Uvinul N539t, 98%, Eurotrading, Padova, Italy) were used as received. *Artemisia arborescens* (L.) essential oil was custom-extracted by steam distillation by Marcello Militello (Ph.D. Agronomist), Regional Institute for Floriculture (IRF) (SanRemo, IM, Italy), in collaboration with “azienda agricola l’essenza degli iblei” (Canicattini Bagni, SR, Italy).

### 3.2. Isolation of Chamazulene (CA)

Chamazulene (CA) was isolated by flash chromatography from *Artemisia arborescens* (L.) essential oil, which contained 13.03% CA by GC-MS. A 2.5 mL quantity of EO was seeded in a flash column prepared with a borosilicate glass heavy-wall chromatography column (18 mm i.d × 400 mm length, Macherey-Nagel) loaded with silica gel (spherical, 60 µm, Merck, Milan, Italy) eluted with *n*-hexane via a manual eluent pumping system. Elution progress was monitored by TLC on glass plates 50 mm × 100 mm (Sigma Aldrich). After elution, fractions having > 99% purity based on GC-MS peak area were combined and evaporated under vacuum to yield 138 mg of CA (purity 99.8% by GC-MS) with an isolation yield of 5.52%.

### 3.3. Photolysis Experiments: Equipment and General Procedures

The photochemical behavior of CA was investigated using a custom-made UV light box with a combined mercury/tungsten light source emitting in the UV-VIS (Osram Ultra-Vitalux^®^, OSRAM GmbH, Munich, Germany). The lamp produces a mix of radiation very similar to that of natural sunlight. This blend of radiation is generated by a high-pressure mercury discharge tube (ultraviolet emission) and a tungsten filament (infrared and visible light emission). Compliant with the technical specification of the lamp, the distance from the sample for photostability experiment was regulated to obtain an irradiance of 2.4 mW/cm^2^ in the UV-A and of 0.5 mW/cm^2^ in the UV-B region; this irradiance is of the same magnitude as the UV exposure from solar irradiation in a summer sunny day at noon across the world (based upon reports of UVA intensity of 2.1 mW/cm^2^ in Okayama, Japan in September [46] and 3.6 mW/cm^2^ in Jackson (MS), USA in August [53]. In the preliminary studies to identify CA fragmentation products the irradiance was raised by shortening the distance of the sample to the lamp so as to achieve about 70 mW/cm^2^ in the UVA. Samples were prepared by simple dissolution of CA in the desired solvent (acetonitrile, *n*-hexane, or methanol) at a concentration of 1.0 g/L for the initial identification of photodegradation products, or of 0.03 g/L to 0.5 g/L for photostability kinetics, as indicated in Section 2.3. When needed, additives (antioxidants or sunscreens) were dissolved in the same solution at the desired concentration (see Section 2.3): samples were placed in Suprasil^®^ quartz EPR tubes (4 mm ID) and sealed. When needed, degassing was performed by bubbling N_2_ for 2 min via a glass capillary. Irradiated samples were then subjected to GC-MS, UHPLC-PDA or UHPLC-ESI-MS^n^ analysis as appropriate. 

### 3.4. GC-MS Analysis

Gas chromatography/mass spectrometry (GC-MS) analyses were performed on a TRACE GC 1310 Series (Thermo Fisher Scientific, Rodano, MI, Italy) gas chromatograph equipped with a split-splitless injector and interfaced with a ISQ QD (Thermo Fisher Scientific) mass detector with single quadrupole analyzer, operating in electron impact (EI) mode. The GC column was a Phenomenex ZB-5 fused-silica capillary column (30 m, 0.25 mm i.d., 0.25 µm film thickness), consisting of crossbond 5% diphenyl, 95% dimethyl polysiloxane. Injection was performed by CombiPal autosampler (CTC Analytics, Zwingen, Switzerland). Helium (He) was the carrier gas at a flow rate of 1.2 mL/min. Two distinct temperature programs were used, the first (method A) used to verify the purity of fractions from flash chromatography and the second (method B) used for rapid analysis of the CA during pho-tostability experiments. Method A: initial temp. 50 °C, held for 2.5 min, then increased at 2 °C/min to 220 °C, for a total run time of 85 min. Method B: initial temp. 120 °C ramped by 5 °C/min to 195 °C, for a total run time of 10 min. The temperatures of the injector base, the transfer line and the ionization source were maintained at 250, 280 and 300 °C, respectively. The mass spectra were recorded in full scan (40–650 amu) to collect the total ion current (TIC) chromatograms. Calibration for CA is shown in Figure S4 (Supplementary Materials).

### 3.5. UPLC-ESI-MS^n^, ESI-MS^n^ Analysis

Ultra-high-performance liquid chromatography (UHPLC) analyses were carried out on a ACCELA liquid chromatograph interfaced with a photo diode array (PDA) detector, with an autosampler and with a LCQ FLEET mass spectrometer (Thermo Fisher Scientific). The mass spectrometer was equipped with an electron spray ionization (ESI) source and with an ion-trap analyzer. The ESI system, operating in positive mode, was set at 6.0 kV and 8 V for spray and capillary voltage, respectively; the heated capillary was at 300 °C. The sheath gas and the auxiliary gas (nitrogen) flow rates were set to 25 and 3 (arbitrary unit), respectively. The ESI process was optimized using pure isolated CA as reference compound. The mass chromatograms were acquired in total ion current (TIC) modality from 50 to 2000 *m/z* and in MS/MS mode (collision-induced dissociation, CID 25, arbitrary) on the protonated molecule of CA and its photoproducts. To identify CA photodegradation products, photo-exposed samples were also infused directly into the ESI interface via 500 µL syringe (Hamilton) and a syringe pump device. The parent ion produced in MS was isolated in the ion-trap (MS^2^) subjected to CID at 25 AU and fragments analyzed (MS^3^). HighChem Mass Frontier 5.0 software was used to generate in silico MS/MS fragmentation of some hypnotized photodegraded products of CA. 

Reversed-phase chromatographic analyses were performed on a Kinetex PFP (Phenomenex), 100 mm × 3.0 mm i.d. × 2.6 µm column. For the initial screening of CA degradation products the following program was used (Method A): flow rate 300 µL/min; injection volume 5 µL; mobile phase was 0.1% aqueous formic acid (solvent A) and 100% acetonitrile (solvent B); linear gradient profile (A:B) started at 40:60, kept for 4 min, then linearly decreased to 5:95 in 30 min, kept constant for 5 min and finally re-equilibrated at 40:60 in 5 min. Total run time was 40 min. 

### 3.6. LC-PDA Analysis

LC- PDA mode was operated to rapidly quantify CA during the course of photolysis kinetics experiments. The apparatus is described in Section 3.5. Spectra of acquisition were from 250 to 800 nm, and a wavelength of 349 nm was selected to monitor CA. Chromatographic separation was achieved with a faster elution program (Method B): flow rate 600 µL/min; injection 2 µL; mobile phase consisted of 0.1% formic acid aqueous (solvent A) and 100% acetonitrile (solvent B). Linear gradient profile (A:B) started at 40:60, was kept constant for 2 min, then linearly decreased to 30:70 in 2 min, then linearly decreased up to 5:95 in 1 min, kept constant for 2 min and finally linearly increased up to 40:60 in 2 min. Total run time was 10 min. Calibration for CA is shown in Figure S5 and typical chromatograms in Figures S6 and S7 (Supplementary Materials).

### 3.7. UV-Vis Spectrophotometry

Kinetics of CA degradation during irradiation in the absence of oxygen were performed with a double beam UV/Visible spectrophotometer Lambda 20 (PerkinElmer, Monza, MI, Italy) Full scan spectra were recorded every 60 min in a wavelength range 200 to 800 nm. Sample was contained in 3.5 mL quartz cuvette, degassed by bubbling N_2_ for 5 min and sealed with a Teflon stopper.

### 3.8. Statistical Analysis

All experiments were performed in duplicate and reported as average ± SD. ANOVA and a Student two-sided *t*-test was used to evaluate the significance of the differences between CA solutions in the presence/absence of stabilizers. Significance was set at *p* > 0.01.

## 4. Conclusions

Chamazulene undergoes extensive degradation when exposed to solar light, with changes to its distinctive color and, conceivably, to its biological properties. Our multi-technique analytical approach, combining GC-MS, UHPLC-PDA-ESI-MS/MS and direct infusion ESI-MS^n^ matched to in silico mass spectral simulations, allowed us to identify 7 degradation products upon exposure of a CA solution to intense (70 mW/cm^2^ UVA) irradiation for 3 h corresponding to a blue-to-green color change. Among them, CA dimers and CA benzenoid are likely responsible for the green color development, since they disappeared upon more extended irradiation (6 h), corresponding to color fading to yellow. This knowledge is relevant in cosmetics science where CA is gaining importance as natural coloring agent. Photostability tests at reduced irradiance under different settings, indicated that the rate of degradation increased with the polarity of the solvent and was highly sensitive to air, becoming much-reduced to negligible in degassed samples. This suggested a photo-oxidative mechanism, likely involving polar transient species including the hydroperoxyl/superoxide (HOO•/O_2_^−•^) radical. Consistent with this conclusion, CA could be significantly spared by antioxidants like hydroxytyrosol and α-tocopherol, yet particularly by TEMPO, which specifically acts by exploiting the formation of HOO• radicals. Sunscreens of common use in cosmetics, i.e., octyl methoxycinnamate and particularly Tinosorb^®^ S could also affectively spare CA photodegradation. However, other tested antioxidants (bakuchiol, ascorbyl palmitate, γ-terpinene) and their combination and other tested sunscreens (octocrylene) were ineffective, likely due to limited stability under the photolysis test conditions—an hypothesis that deserves further investigation. Surprisingly, CA was perfectly stable in the dark at 50 °C, showing however reduced stability upon incubation with ascorbyl palmitate, which implies that the purported limited stability in formulated products (e.g., cosmetics) is likely due to facile reaction with byproducts formed upon decomposition of other components. Although none of the tested protection strategies alone afforded complete inhibition of CA photodegradation, we believe that current results provide a solid basis for successful developments in that direction.

## Figures and Tables

**Table 1:** MS^n^ fragmentation data of chamazulene and its photodegradation products, obtained by collision-induced dissociation (CID). RA: Relative abundance.

Peak #	Compound	Retention Time (min)	Parent Peak *m/z* [M + H] ^+^	Main Product Ion	RA (%)
**2**	Oxidized chamazulene	2.88	183	169 [M + H − CH_2_]^+^	48
				155 [M + H − CH_2_=CH_2_]^+^	38
				141 [M + H − C_3_H_6_]^+^	35
**3**	Chamazulene quinone	3.61	215	197 [M + H − H_2_O]^+^	40
				187 [M + H − CO]^+^	100
				173 [M + H − CH_2_CO]^+^	33 38
				159 [M + H − CH_2_=CH_2_ − CO]^+^	
**4,5**	Chamazulene carbaldehyde	3.75	199	184 [M + H − CH_3_]^+^	25
		3.85		171 [M + H − CO]^+^	100
				143 [M + H − CH_2_=CH_2_ − CO]^+^	78
**6**	Dihydrochamazulene	4.44	187	172 [M + H − CH_3_]^+^	15
				159 [M + H − C_2_H_4_]^+^	100
				131 [M + H − C_4_H_8_]^+^	22
**7**	Chamazulene benzenoid	5.79	399	381 [M + H − H_2_O]^+^	100
				371 [M + H − CO]^+^	57
				215 [M+H − Chamazulene]^+^	10
				199 [M+H − CH_3_Chamazulene] ^+^	63
**8**	Chamazulene	7.89	185	169 [M + H − CH_4_]^+^	100
				129 [M + H − CH_2_=CHCH_2_CH_3_]^+^	10
**9**	Methylene dimers of chamazulene	13.06	381	366 [M + H − CH_3_]^+^	38
				363 [M + H − H_2_O]^+^	100
				353 [M + H − CH_2_=CH_2_]^+^	22
		14.90		337 [M + H − CH_2_=CHCH_3_]^+^	30
				321 [M + H − C_6_H_6_]^+^	33
**10**	Chamazulene dimers	16.51	367	352 [M + H − CH_3_]^+^	60
		17.05		338 [M + H − CH_3_CH_2_]^+^	100
		18.28		309 [M + H − C_4_H_10_]^+^	25

**Table 2:** Radiometric values used for all samples subjected to photostability tests (T = 25 °C).

Type of UV	Wavelength [nm]	Radiance [mW cm^−2^ sr^−1^]	Irradiance [mW cm^−2^]	Energy Absorbed [J cm^−2^] (t = 15 min)	Energy Absorbed [J cm^−2^] (t = 4 h)
UVA	320–400	35.2	2.4	2.2	34.6
UVB	290–320	7.5	0.5	0.5	8

**Table 3:** Kinetics of CA photodegradation under irradiation conditions detailed in Table 2, as a function of the solvent and the degree of air exposure and head space volume (HS). Regressions are expressed as residual CA% (y) as a function of time in minutes (x).

Air Saturated			
	HS 0 cm^3^	HS 0.17 cm^3^	HS 0.34 cm^3^
*n*-Hexane	y = −0.0551x + 99.638 *R*^2^ = 0.9368	y = −0.0654x + 100.94 *R*^2^ = 0.9958	y = −0.0736x + 101.04 *R*^2^ = 0.9692
Methanol	y = −0.1107x + 101.17 *R*^2^ = 0.9659		
Acetonitrile	y = −0.3173x + 102.56 *R*^2^ = 0.9811		
**N_2_ purged**			
	**HS 0 cm^3^**		
*n*-Hexane	y = −0.0009x + 100.72 *R*^2^ = 0.0033		
Methanol	y = −0.0417x + 101.75 *R*^2^ = 0.7921		
Acetonitrile	y = −0.1146x + 103.17 *R*^2^ = 0.8943		

**Table 4:** Comparative rate of photodegradation of 500 mg/L CA in acetonitrile (100%) irradiated at 2.4 mW/cm^2^ at 25 °C, alone or in the presence of different antioxidants or of different sunscreens (* = significantly different from the corresponding value for irradiated unprotected CA; *p* > 0.01). The time-course is expressed as cumulative irradiation dose.

UV Irradiated Solutions of CA	Percentage of CA after UVA Irradiation						
	0 J/cm^2^	2 J/cm^2^	4 J/cm^2^	8 J/cm^2^	17 J/cm^2^	25 J/cm^2^	35 J/cm^2^
CA	100	98 ± 1	92 ± 2	81 ± 2	52 ± 3	22 ± 1	0
***MIXED WITH ANTIOXIDANTS***							
CA + TEMPO (1:10)	100	96 ± 3	90 ± 2	83 ± 4	73 ± 3 *	53 ± 1 *	33 ± 1 *
CA + Tocopherol (1:10)	100	94 ± 2	88 ± 2	77 ± 1	65 ± 2 *	43 ± 1 *	27 ± 1 *
CA + Ascorbyl palmitate (1:10)	100	95 ± 2	94 ± 4	71 ± 3	49 ± 3	2 ± 1 *	0
CA + Bakuchiol (1:10)	100	94 ± 3	91 ± 3	62 ± 3 *	11 ± 3 *	0 *	0
CA + Hydroxytyrosol (1:10)	100	94 ± 3	92 ± 3	80 ± 2	62 ± 2 *	37 ± 1 *	6 ± 1 *
CA + Tocopherol + Bakuchiol (1:1:1)	100	94 ± 1	92 ± 1	77 ± 3	41 ± 1 *	10 ± 1 *	0
CA + Ascorbyl palmitate + Tocopherol (1:1:1)	100	91 ± 2	80 ± 2 *	68 ± 3 *	17 ± 1 *	0 *	0
CA + Tocopherol + Hydroxytyrosol (1:1:1)	100	93 ± 2	81 ± 4	72 ± 3 *	37 ± 2 *	8 ± 3 *	0
CA + Tocopherol +TEMPO (1:1:1)	100	96 ± 3	90 ± 2	76 ± 1	19 ± 1 *	0 *	0
CA + Ascorbyl palmitate + TEMPO (1:1:1)	100	96 ± 3	90 ± 2	75 ± 2	17 ± 1 *	0 *	0
CA + γ-terpinene + Ascorbyl palmitate (1:5:1)	100	94 ± 2	92 ± 3	76 ± 2	28 ± 4 *	0 *	0
CA + γ-terpinene + Hydroxytyrosol (1:5:1)	100	96 ± 2	90 ± 3	78 ± 1	38 ± 2 *	0 *	0
CA + γ-terpinene + TEMPO (1:5:1)	100	93 ± 1	93 ± 2	80 ± 2	57 ± 2	24 ± 1	6 ± 1 *
***MIXED WITH UV FILTERS***							
CA + Tinosorb^®^ S (5%)	100	92 ± 2	92 ± 3	86 ± 2 *	73 ± 4 *	65 ± 2 *	48 ± 1 *
CA + Octocrylene (5%) ^¥^	100	98 ± 3	90 ± 2	79 ± 3	57 ± 1	22 ± 1	0
CA + Octyl methoxycinnamate (5%)	100	100 ± 1	99 ± 3	97 ± 3 *	70 ± 3 *	45 ± 2 *	20 ± 2 *
CA in the dark (negative control)	100	100 ± 2	99 ± 2 *	99 ± 2 *	100 ± 1 *	99 ± 3 *	100 ± 2 *

^¥^ All tested additives produced a significantly different (*p* < 0.01) overall response to irradiation as compared to unprotected CA, except octocrylene, according to two-sided ANOVA analysis.

## Data Availability

The data presented in this study are available on request from the corresponding author.

## Supplementary Materials

The following supporting information can be downloaded at: https://www.mdpi.com/article/10.3390/molecules29112604/s1, Figures S1–S9: GC-MS analysis of *M. chamomilla*, *A. millefolium* and *A. arborescens*, spectral distribution of the solar lamp, fragmentation of ion *m/z* 215, calibration curves for GC-MS and LC-PDA analysis, example of chromatograms decay of CA irradiated in the absence of oxygen and in the dark in the presence of antioxidants.
