# Supplementary material for: Photochemical and Oxidative Degradation of Chamazulene Contained in Artemisia, Matricaria and Achillea Essential Oils and Setup of Protection Strategies
Source: Molecules. 2024 Jun 1;29(11):2604. doi: 10.3390/molecules29112604 (PMC11173868; doi:10.3390/molecules29112604)
Supplement: Supplementary file 1 [file molecules-29-02604-s001.zip › molecules-3021095-supplementary.pdf]

# Photochemical and oxidative degradation of chamazulene contained in *Artemisia, Matricaria* and *Achillea* essential oils and setup of protection strategies

Simone Gabbanini,*[,1] Jerome Ngwa Neba,[2] Riccardo Matera,[1] Luca Valgimigli, *[,2,3]

[1] R&D Department, BeC s.r.l., Via C. Monteverdi 49, 47122 Forlì, Italy; laboratorio@bec-natura.com

[2]Department of Chemistry "Ciamician", University of Bologna, Via Gobetti 85, 40129 Bologna, Italy; luca.valgimigli@unibo.it

[3] Tecnopolo di Rimini, Via D. Campana 71, 47922 Rimini, Italy.

APPENDIX

(Supplementary data)

Table of Contents


| | |
|---|---|
| **Figure S1.** GC-MS analysis of *A. arborescens*, *M. chamomilla* and *A. millefolium* EOs. | Page 2 |
| **Figure S2.** Spectral radiation power distribution of Ultra-Vitalux lamp | Page 2 |
| **Figure S3.** Fragmentation of parent peak *m/z* 215, calculated by Mass Frontier software | Page 3 |
| **Figure S4**. GC-MS calibration curve of CA in Total Ion Count (TIC) mode | Page 4 |
| **Figure S5**. LC-PDA calibration curve of CA at 349 nm | Page 4 |
| **Figure S6**. Example of LC-PDA chromatograms of mixtures of CA and antioxidants. | Page 5 |
| **Figure S7**. Example of LC-PDA chromatograms of mixtures of CA (0.17 mM) with UV filters | Page 6 |
| **Figure S8.**Spectrophotometric evaluation of CA photodegradation in the absence of oxygen | Page 7 |
| **Figure S9.** Detail of chamazulene degradation with selected antioxidants, incubated at 50°C. | Page 7 |

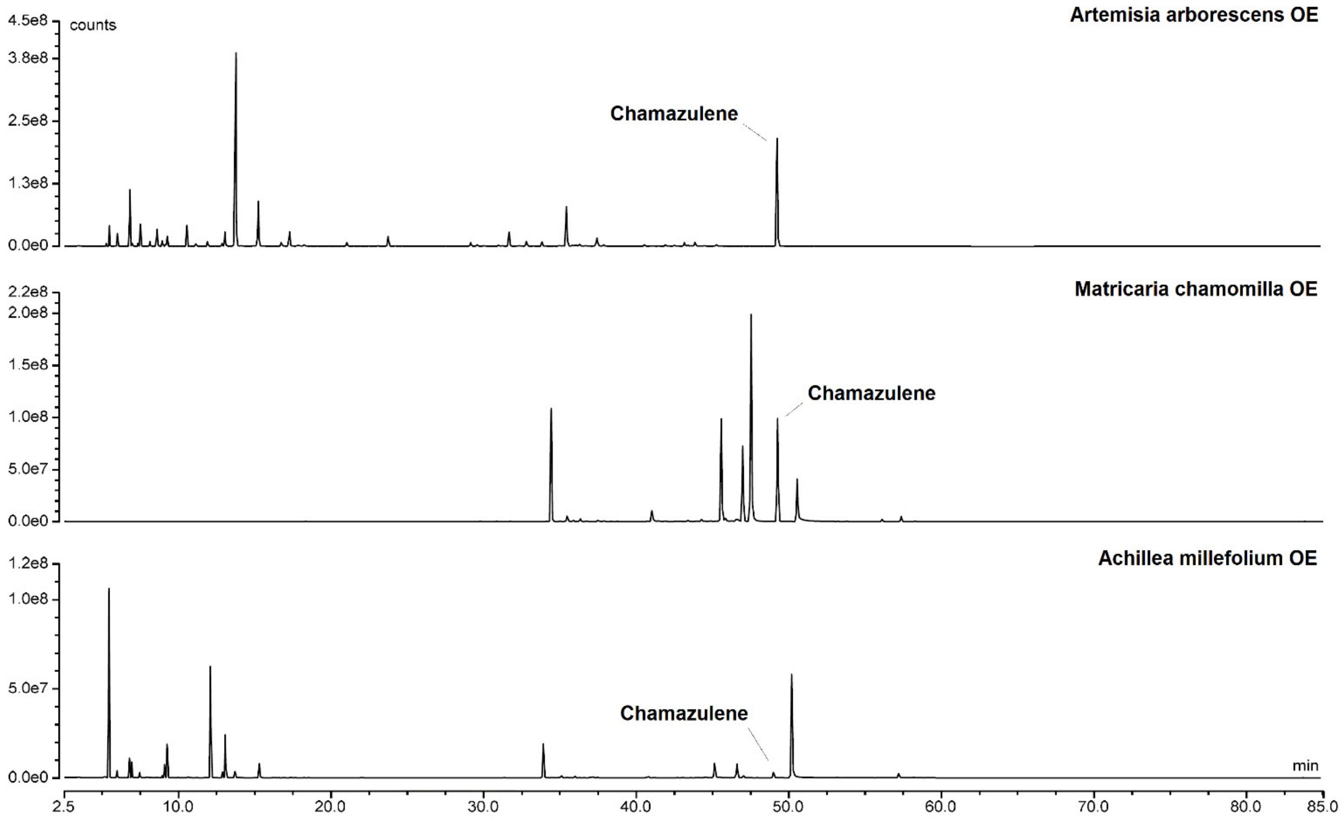


**Figure S1.** GC-MS analysis of three representative EO samples of *A. arborescens*, *M. chamomilla* and *A. millefolium*, comparatively screened to identify the most convenient source of chamazulene for isolation. The respective chamazulene content was 13.6%, 9.6% and 1.2% by peack area in TIC chromatograms.

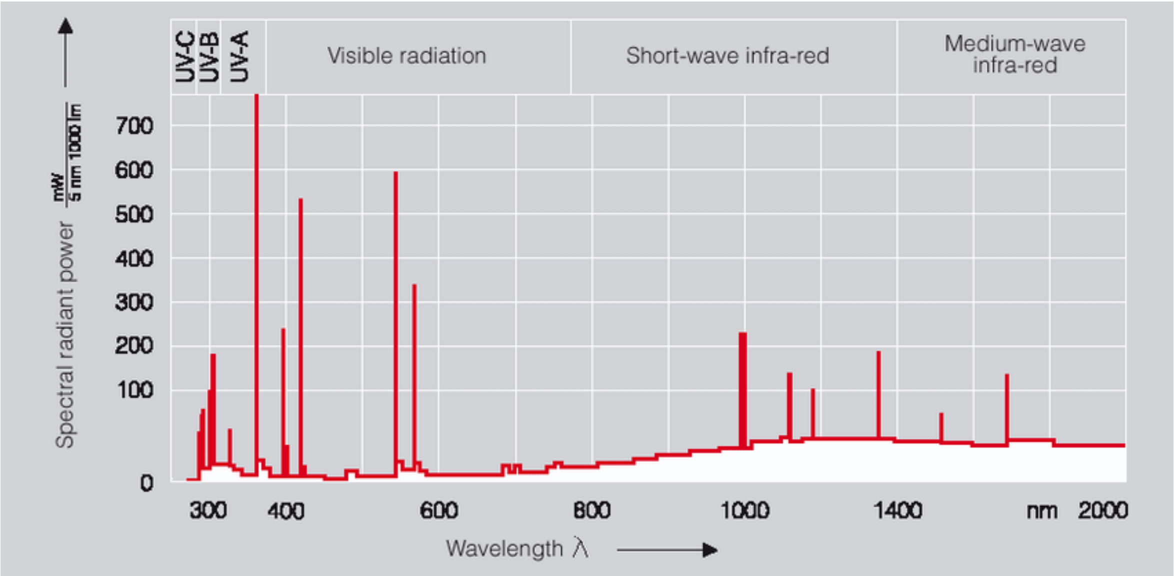


**Figure S2.** Spectral radiation power distribution of Osram Ultra-Vitalux® lamp at various wavelengths. Data provided by the lamp manufacturer, reported only as a reference.

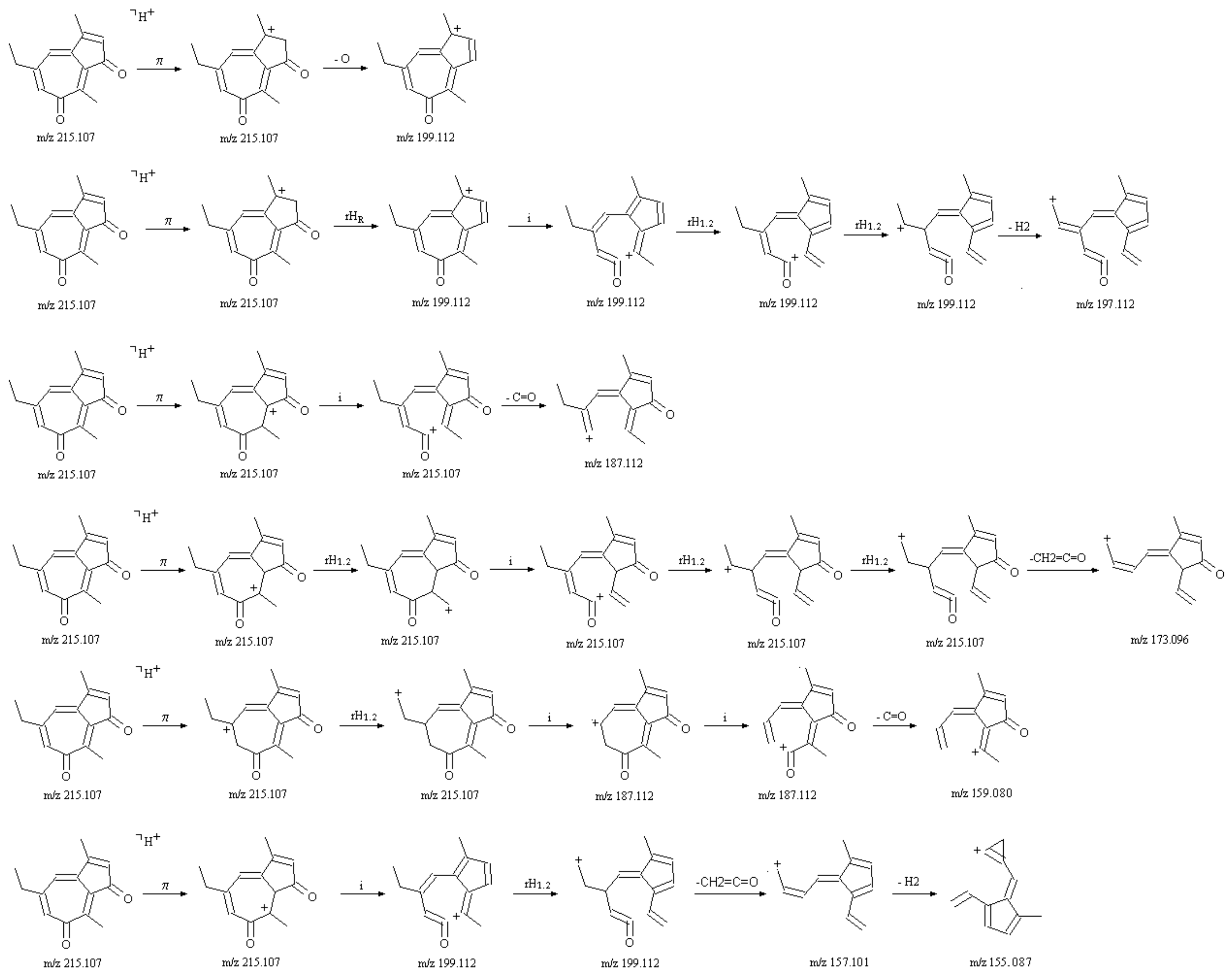


**Figure S3.** Fragmentation ions of parent peak *m/z* 215, in positive mode attributed to chamazulene quinone (6), calculated by Mass Frontier software. The fragmentation pattern explains ions with *m/z* 197, 187 and 159 shown in Figure 9.

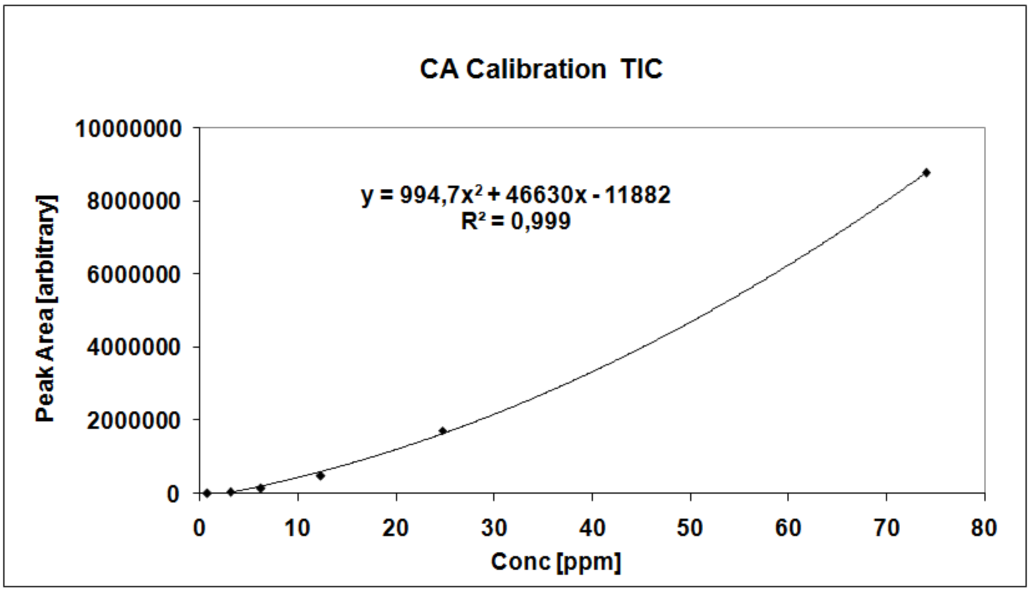


**Figure S4**. GC-MS calibration curve of CA in Total Ion Count (TIC) mode, used to evaluate the influence of solvent type and presence of oxygen on the photostability of CA alone.

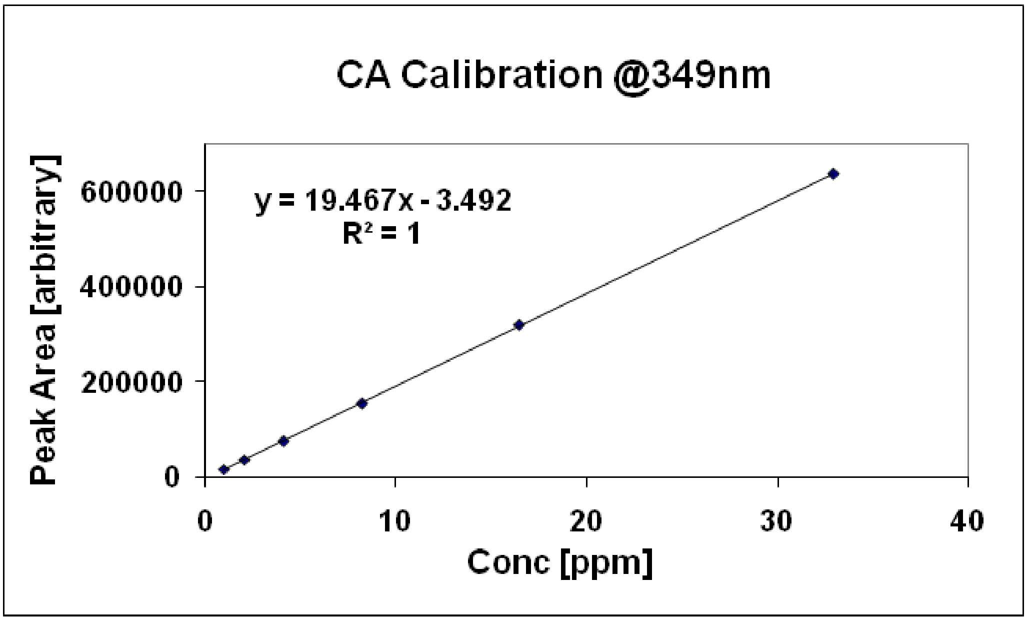


**Figure S5**. LC-PDA calibration curve of CA at 349 nm used for evaluation of photo and thermal stability experiments of CA in presence of antioxidants and sunscreens.

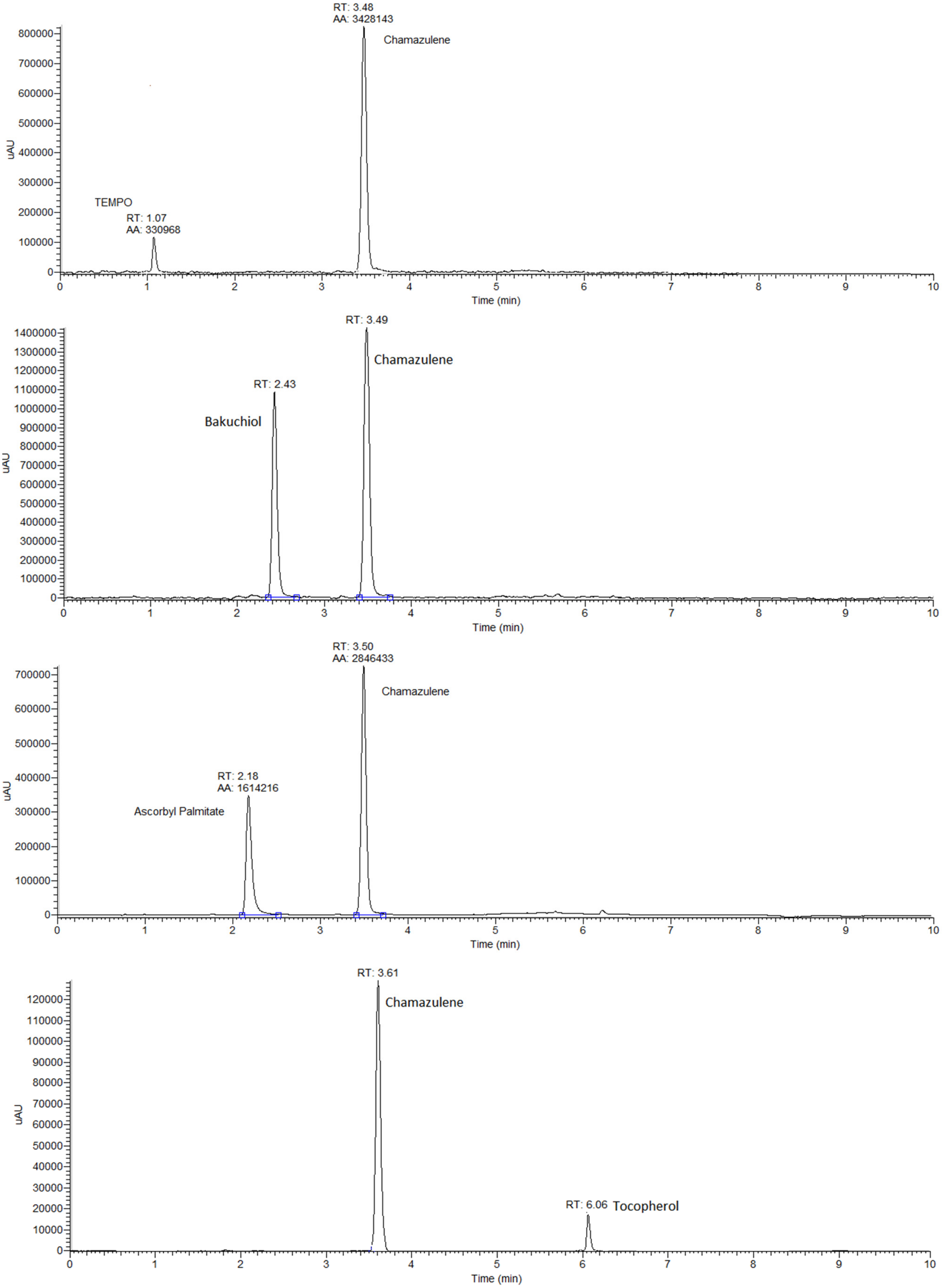


**Figure S6**. Example of HPLC-PDA chromatograms of mixtures of CA:antioxidants in molar ratio 1:10. From top to bottom: TEMPO, bakuchiol, ascorbyl palmitate, tocopherol.

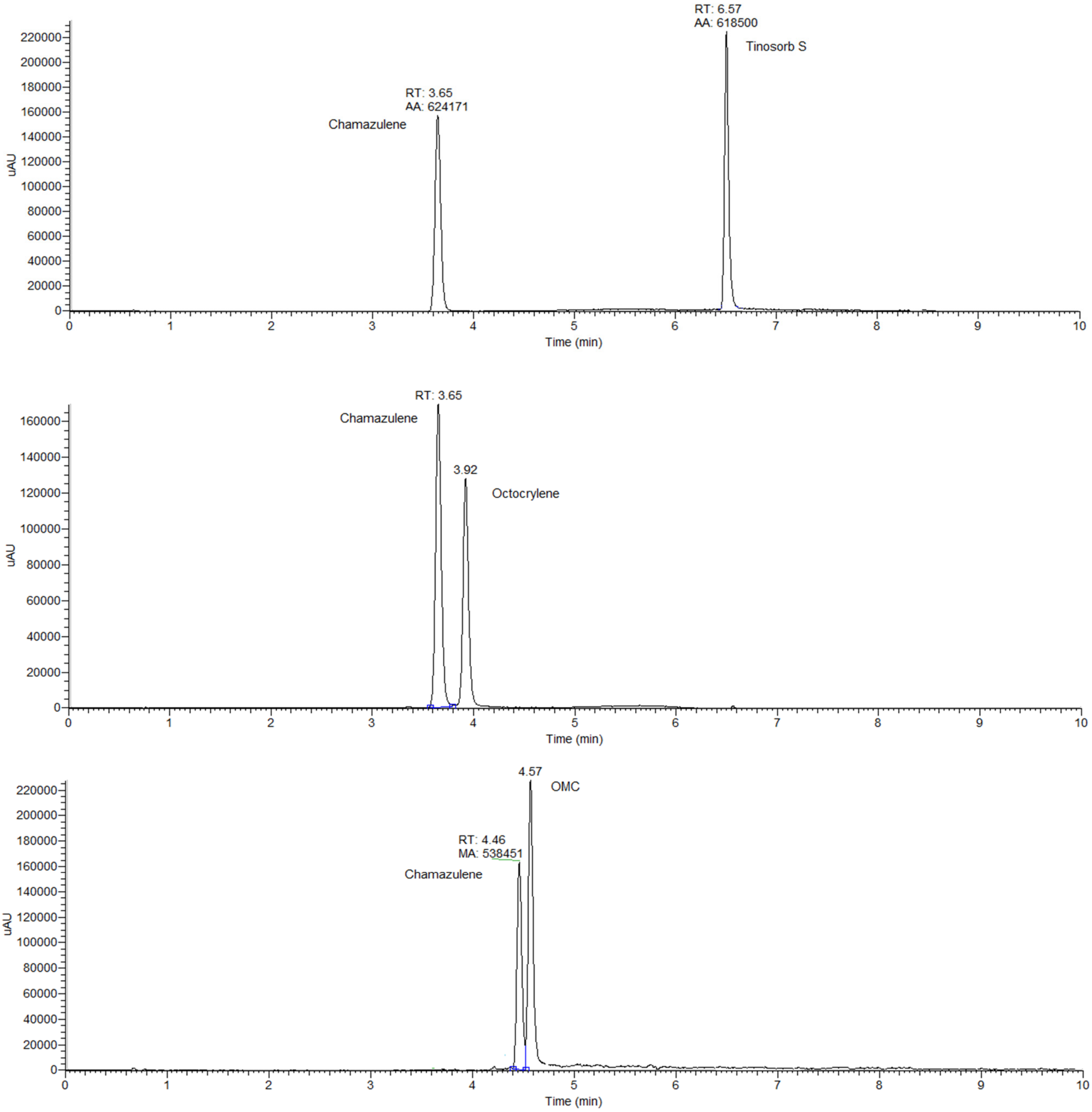


**Figure S7**. Example of LC-PDA chromatograms of mixtures of CA (0.17 mM) with UV filters at a concentration of 5% w/v (from top to bottom:, Tinosorb® S, octocrylene, octyl methoxycinnamate). The choormatographic conditions were slightly changed for octlyl methoxycinnamate to resolv coelution with chamazulene..

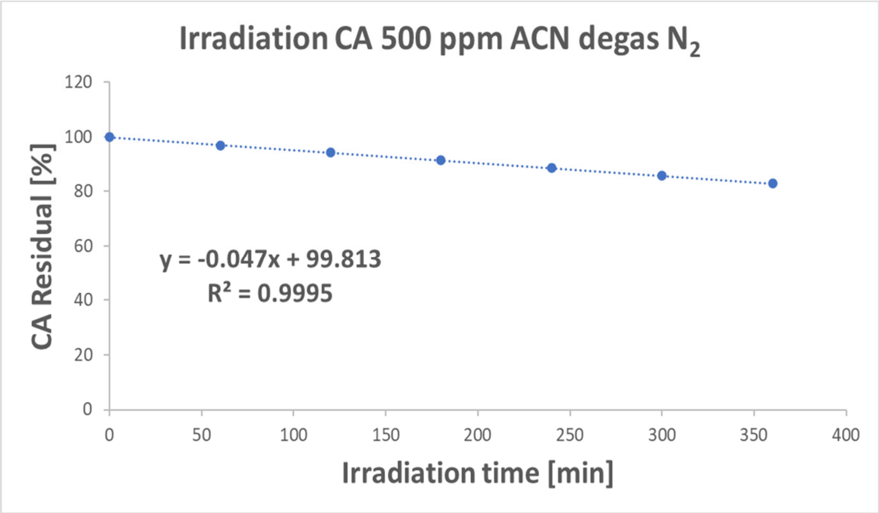


**Figure S8.**Spectrophotometric evaluation of CA photodegradation in the absence of oxygen. Measurments were perfomed in a sample of CA 500 ppm in acetonitrile in a 3.5 mL Teflon sealed quartz cu-vette and degassed for 5 minutes with an abundant $N_2$ stream. The cuvette was subjected to UVA-UVB radiation at100 mW/cm$^2$ (strong photooxidation conditions)for 6 hours. At regular time intervals a spectrophotometric reading was performed without opening the cuvette. The rate of the CA degradation is reported.

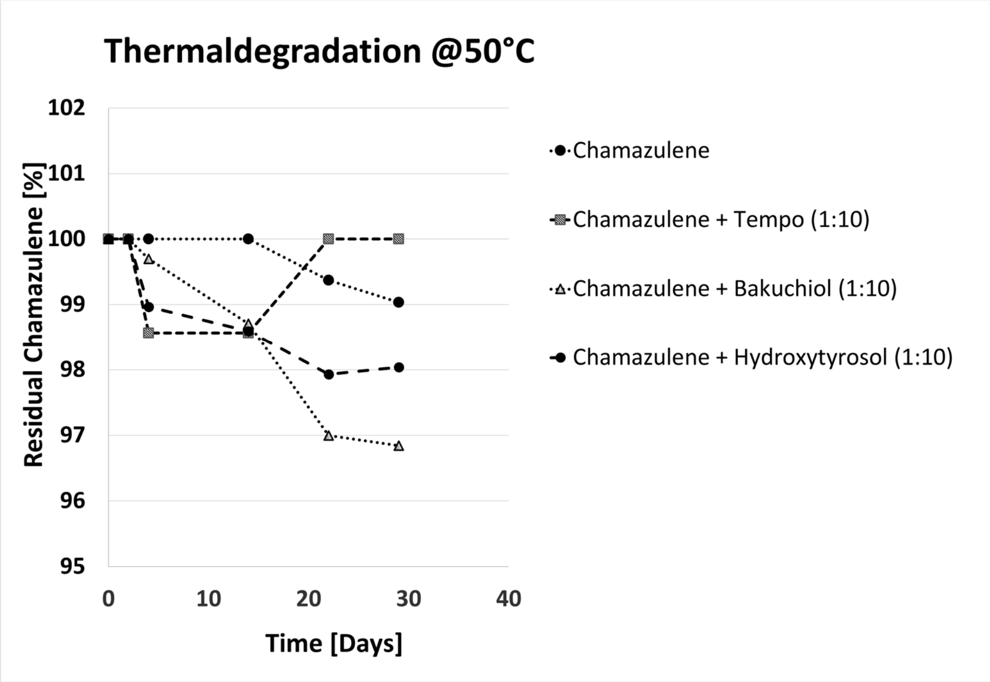


**Figure S9**. Detail of thermal degradation of chamazulene in solution, alone or in the presence of selected antioxidants, incubated at 50°C. This figure represents and expansion of Figure 12 in the manuscript.
